# A Plasmonic based Ultracompact Polarization Beam Splitter on Silicon-on-Insulator Waveguides

**DOI:** 10.1038/srep02206

**Published:** 2013-07-16

**Authors:** Qilong Tan, Xuguang Huang, Wen Zhou, Kun Yang

**Affiliations:** 1 Laboratory of Nanophotonic Functional Materials and Devices, South China Normal University, Guangzhou, 510006, China

## Abstract

An ultracompact polarization beam splitter (PBS) is designed on silicon-on-insulator (SOI) platform based on the localized surface plasmons (LSPs) excited by particular polarization light. The device uses nanoscale silver cylinders as the polarization selection between two silicon waveguides of a directional coupler. The transverse-magnetic (TM) polarization light excites localized surface plasmons and is coupled into the cross port of the directional coupler with a low insert loss, while the transverse-electric (TE) polarization light is under restriction. The PBS has a coupling layer with 50 nm width and 1.1 μm length supporting broadband operation. The simulation calculations show that 22.06dB and 23.06dB of extinction ratios for the TE and TM polarizations were obtained, together with insertion losses of 0.09dB and 0.40dB.

Future development of integrated optics is expected to rely on utilization of well-established complementary metal-oxide-semiconductor (CMOS) fabrication technologies that have been proven to facilitate high-volume manufacturing of highly integrated electronic devices and systems^1^. As an important integrated optics device^2^, a compact PBS can be used to achieve polarization independent operation of photonic integrated circuits (PICs) and linear optical quantum information technology^3^. A perfect ultracompact PBS should simultaneously have short device length, high extinction ratios, low insertion loss, broadband operation, stability, simple structure and high fabrication tolerances. Although immense amount of concrete researches have been done, few perfect ultracompact PBS has been reported.

There are two main methods to realize polarization splitting: adiabatic mode evolution and mode coupling. The adiabatic designs usually require large device footprint because of the adiabatic operation restrained, for example, the report of Watts et al used a 200 μm long device to achieve extinction ratio of 22 dB^4^. In comparison, mode coupling based devices can be much shorter with coupling lengths of a few micrometers, but usually suffer from smaller bandwidths and tighter fabrication tolerances^5^,^6^, and thus the latter shows more attraction.

A mode coupling based PBS needs some source of birefringence in the structure; the waveguide birefringence directly determines the polarization splitting ratio of the device. The devices consisting of photonic crystals^7^, asymmetric structures^8^, slot waveguides^9^, and bent directional coupler^10^ have been reported, and some propositions have been demonstrated by experiments. However all the designs above have small bandwidths problem and others.

The large birefringence of a metal provides another method. Surface plasmon polaritons (SPPs)^11^ are excited by specific polarization light. The specific polarization light has a much shorter coupling length than the orthogonal polarization, and it can reduce the coupling length. The C.-L. Zou^12^ proposed a broadband integrated waveguide polarization beam splitter consisting of a metal nanoribbon and two dielectric waveguides. Then a PBS utilizing a 6.5 μm long hybrid plasmonic waveguide as the middle waveguide in the three-core arrangement^5^ experimentally demonstrated the large birefringence achieved by SPPs. We summarize the former experience and propose a novel PBS based on the LSPs. The LSPs do not require wave vector matching as the excitation of SPPs, and it has high excitation efficiency^13^. These characters can further reduce the coupling length and insertion losses.

The device showing in Figure 1. consists nanoscale silver cylinders sandwiched between two silicon waveguides on a silica substrate. The diameter of cylinders is *D*, the edge-to-edge separation between adjacent cylinders is *d*, the width of the silicon waveguide is w, and the length of the coupling region is *L*. The coupler is aimed to operate in the optical communication wavelength of 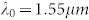. As a noble metal material, silver provides relatively low propagation losses and stable chemical properties. The high refractive index contrast of the material 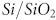 allows for forceful confinement of guided modes. Currently, the refractive indices in integrated optics are usually chosen to be 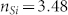 and 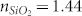
14.

## Results

**Figure 2:**
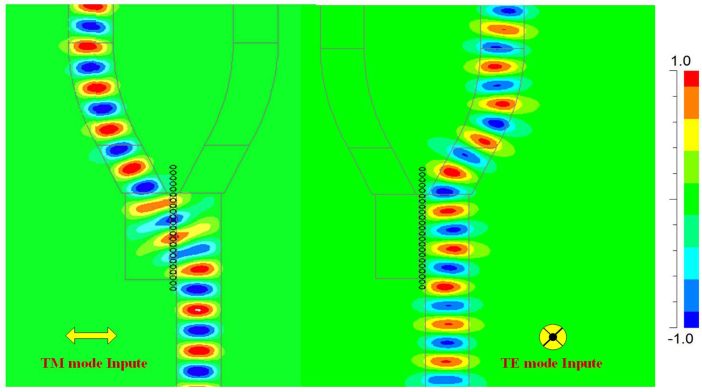
The light intensity distributions of the TM and the TE modes, the different polarization light propagates in the respective path.

According to the proposed structure, the power transmission from input port to each mode export is computed. Figure 2. shows the propagations of the TE and TM polarization modes at communication wavelength 1.55 μm. One can observe that there is obvious polarization selectivity with low reflections.

The key performance characteristics of the PBS include: extinction ratios (ERs), insertion losses (ILs), and 15 dB bandwidths. The extinction ratios of the PBS are defined as 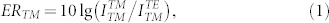

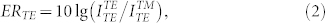
where 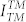 is the power of TM mode at the TM port, and 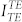 is the power of TE mode at the TE port, 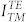 is the power of TE mode at the TM port, 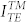 is the power of TM mode at the TE port. The 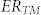 and 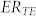 respectively signify the extinction ratios of the different mode port.

The insertion losses of different modes are defined as 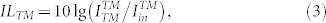

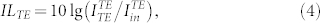
where 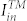 is the power of TM mode at input port, 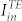 is the power of TE mode at input port.

**Figure 3:**
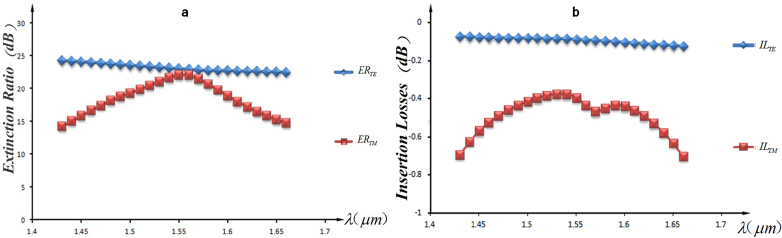
(a) The spectra of extinction ratios. The peak value of 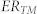 appears at 1.55 *μ*m. (b) The spectra of insertion losses. The curve shows the lowest 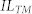 at 1.53 *μ*m.

The simulated results in Figure 3. show the broadband response of the PBS in optics communication wavelengths. The extinction ratios of the TE and TM polarizations are respectively 22.06dB and 23.06dB, with the corresponding insertion losses of 0.089dB and 0.396dB at the length of 1.55 μm. The 15dB bandwidth is 210 nm obtained from Figure 3(a), it is large enough to cover the communication band, and it will preserve 150 nm even if the insertion losses are demanded less than 0.5dB.

## Discussion

Ultracompact devices usually suffer from tight fabrication tolerances, which may cause low rate of finished products. The performance characteristics of the structure are expected to be influenced by the following 4 parameters: (a) the edge-to-edge separation *d* between adjacent cylinders; (b) the diameter of cylinders *D*; (c) the width of the silicon waveguide *w*; (d) the length *L* of the polarization coupling region. One can use the difference 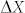 in which both 15dB extinction ratios and 1dB insertion losses can be still maintained to judge the fabrication tolerances. The difference between the maximum and the minimum change is defined as 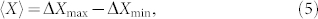
the 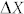 represents the error or the change of parameter *X*.

**Figure 4:**
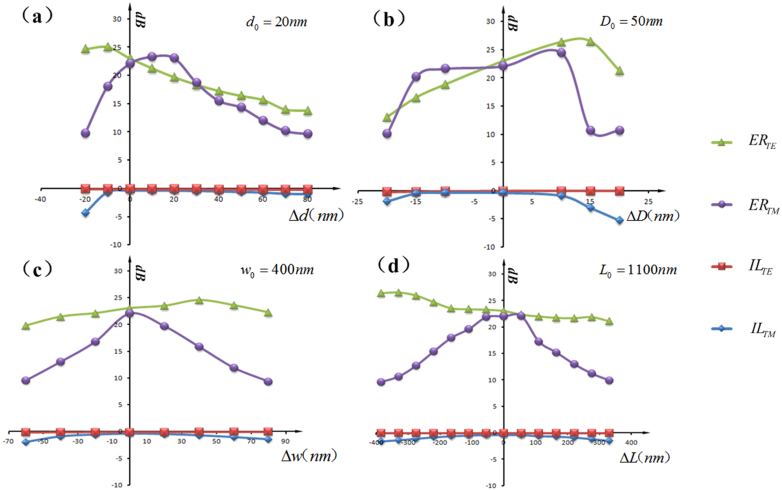
The performance characteristics under the different of parameters. 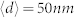
, 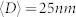, 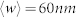 and 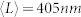.

Figure 4 shows the performance characteristics under the changes of four parameters. The insertion losses of the PBS depend on the energy transfer ratio between the two dielectric waveguides and the absorption loss of silver. The loss can be controlled in a low level due to the ultracompact structure and its optimization. Obviously, the coupler- based PBS may have a few coupling periods, but the optimal length *L* of the coupling region should equal to the semi-period coupling length *L_c_* of the TM polarization. The performance characteristics will turn worse, while the deviation between *L* and *L_c_* increases as the showing of Figure 4(d). The *L_c_* mainly depends on the coupling efficiency from the input waveguide to the cross-port waveguide, the total excitation strength of LSPs, and the LSPs interaction factor between adjacent cylinders. The coupling efficiency mainly depends on the parameters of *D* and *w*, while the total excitation strength of LSPs mainly depends on the LSPs resonance strength (at or near resonance) and the percentage of silver at a fixed length *L*, which is relative to the parameters of *D* and *d*. The LSPs interaction factor between adjacent cylinders mainly depends on the parameter of *d*. Therefore, different parameters will play the leading roles on the performance of the device in different variation ranges.

The parameter *d* has influence on the LSPs interaction factor between adjacent cylinders and the total excitation strength of LSPs, as mentioned. When the *d* increases greater than 40 nm, the LSPs interaction between adjacent cylinders is weak enough to ignore, and the total excitation strength of LSPs will decrease duo to the decrease of percentage of silver. At the same time the LSPs cylinder region can not effectively prevent the TE field from penetrating into the left waveguide. It will cause the decrease of extinction ratios and the increase of *L_c_*. When the separation *d* becomes narrow, the total excitation strength of LSPs will increase. But the LSPs interaction factor between adjacent cylinders will become stronger rapidly, especially when the *d* is less than 20 nm, and the energy will transfer along the array of lossy silver cylinders much more easily. It will lead to large *L_c_* and low extinction ratio for TM mode, which Figure 4. (a) implies.

The parameter *D* directly determines the coupling efficiency between the two dielectric waveguides and the total excitation strength of LSPs. When the *D* turns great enough, the coupling efficiency (due to the increase of the waveguide separation) and the total excitation strength (due to the detuning of the LSPs resonance) will reduce quickly, which leads to rapidly increases of *L_c_* of the TM mode and the insertion loss of *IL_TM_* as well as drops in extinction ratios. When the *D* turns small, the coupling efficiency increases, but the total excitation strength decreases because of the detuning and the decrease of percentage of silver, which results in a balance for the TM polarized light within the range of *D* from 35 nm to 50 nm in Figure 4(b). It is profitable for the fabrication tolerance.

The parameter *w* mainly influences the coupling efficiency and insertion losses. When the *w* reduces, the coupling efficiency and the insertion losses will turn high. If we reduce the *w* to 300 nm, the PBS just needs a 750 nm coupling length, and the extinction ratios of the TE and TM polarizations are respectively 20.69dB and 20.33dB, with the corresponding insertion losses of 0.249dB and 0.671dB. This is the first time that the *L_c_* reduces to the nanoscale for PBS on SOI platform as we know.

The results indicate that the fabrication tolerance of each parameter should be large enough for the existing manufacturing processes^15^,^16^. A novel ultracompact polarization beam splitter on silicon-on-insulator platform based on the LSPs has been proposed and numerically demonstrated. The device can simultaneously maintain high extinction ratios and low insertion losses with broadband operation. It has very short coupling length, stability, simple structure and relatively high fabrication tolerances.

## Methods

**Figure 5:**
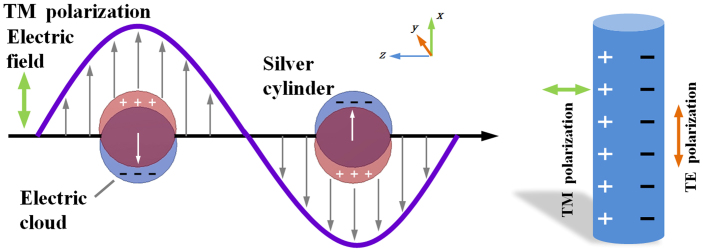
The excitation of LSPs, The conduction electron charge cloud appear displacement relative to the core under the excitation of TM polarization electric filed.

For the case of localized surface plasmons, light interacts with particles much smaller than the incident wavelength, this leads to a plasmon that oscillates locally around the nanoparticle with a resonant frequency known as the LSPR^17^,^18^. An electric field excites uneven distribution of the conduction electrons, this result in strong light scattering, in the appearance of intense surface plasmon absorption bands, and an enhancement of local electromagnetic field^13^. Figure 5. shows the excitation of LSPs. Just the electric field which perpendicular to the surface of a silver cylinder can induce the displacement of the conduction electron charge cloud relative to the core. This means the electric field parallel to the surface of silver cylinders can not excite LSPs, which leads to a large birefringence.

The dielectric constants 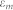 of silver can be calculated by the Drude model^19^,^20^. 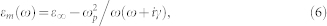
the value of the dielectric constant at infinite angular frequency is 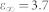, the bulk plasma frequency which represents the natural frequency of the oscillations of free conduction electrons is 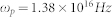, and the damping frequency of the oscillations is 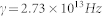, 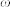 represents the angular frequency of the incident electromagnetic radiation. The nanostructured silver will cause higher losses.

**Figure 6:**
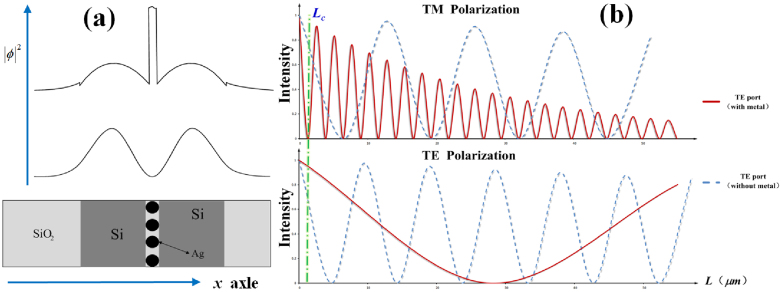
(a) The normalized energy distribution on the x axle in the time average for different polarizations. (b) The energy transfer in the TE port waveguide with and without silver cylinders with the same parameters.

The nanoscale silver cylinders sandwiched between two silicon waveguides will provide a big difference of coupling period for different polarizations due to the large difference in the LSPs excitation between TM and TE fields. Denoting the fields in two separated waveguides with silver cylinders as 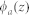 and 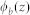, the coupled mode equations^21^ indicate as 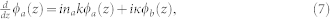

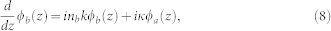
where 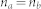 are the effective propagation indices of the waveguides, 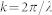 is the wave number, 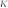 is the coupling efficiency, and have the following relation 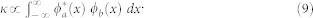
The integral 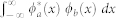 has obvious difference for the different polarizations, which the Figure 6(a) implies. The coupling period has the relation of 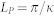, thus the coupling period of TM mode should be shorter than that of the TE mode with the silver cylinders.

Figure 6(b) shows the energy output at TE Port versus the length of L. The oscillations imply the energy exchange between the two silicon waveguides and give the coupling period 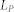. The coupling period of TM mode (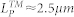) is much shorter than that of the TE mode (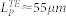) with the silver cylinders sandwiched between two silicon waveguides. Thus, it is easy to separate the two orthogonal polarization modes at about the semi-period region (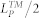) of TM mode. While for the traditional direct coupled waveguides without the silver cylinders, the 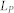 values of TM and TE mode are close to each other, it means a long coupling region (dozens of micron) will be required to separate the two orthogonal polarization.

When light is incident into the PBS from its right dielectric waveguide, the TM component of the light will excite the LSPs on the surfaces of discrete silver cylinders. Because both field enhancements from the nanoscale confinement (in space-domain) and from the resonance (in time-domain) happen, and the excitation of LSPs does not require wavevector matching as the excitation of SPPs, highly efficient excitations of LSPs and strong couplings of the TM field in the right dielectric waveguide (and the TM field in the left dielectric waveguide) with the LSPs can be established, which gives rise to effective energy transferring from the right waveguide into the left waveguide, and backwards in next coupling semi-period. By this way, the coupling between the left and the right waveguides for the TM polarization can be greatly enhanced, with a short coupling length. On the contrary, the TE component of the incident light can not excite the LSPs, and will be hardly coupled into the left waveguide of the PBS, because the forceful confinement and the insulation of the silver cylinders prevent the TE component from penetrating into the left waveguide. Finally, TM and TE components of the incident light will be respectively exported from the left and the right waveguides of the PBS. There may be a little direct coupling between the left and the right waveguides leaking from the gaps between discrete silver cylinders, for each polarization. To achieve high extinction ratios, the direct coupling for the TE polarization should be very little.

## Author Contributions

Q.L.T. contributed to the preliminary scheme, theoretical analysis, simulation, interpretation, and writing. W.Z. and K.Y. contributed to partial data collection. X.G.H. Provide theoretical guidance. All authors discussed the results.

## Figures and Tables

**Figure 1:**
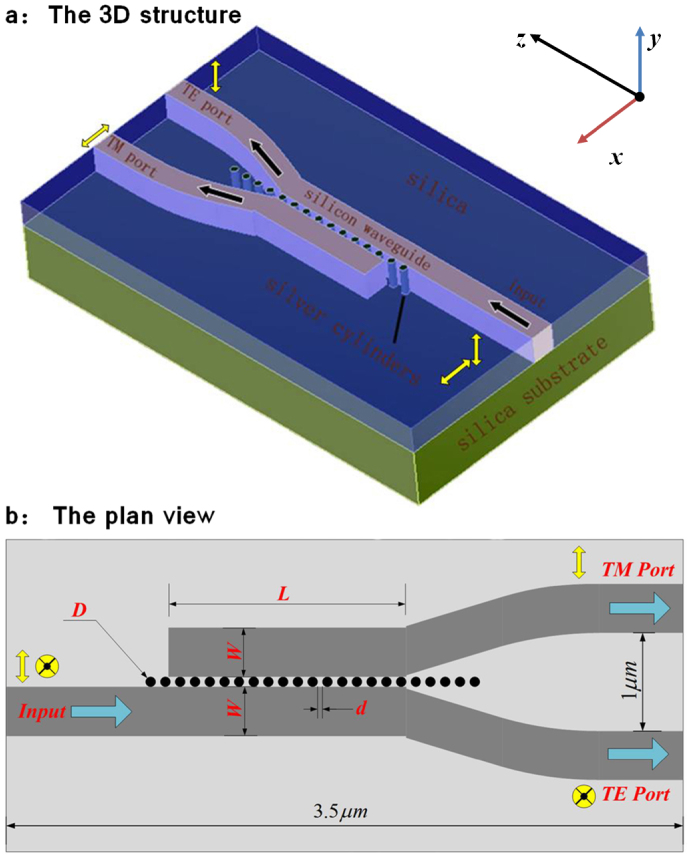
(a) The 3D structure. (b) The plan view of the PBS. The TE and TM polarized lights output at corresponding exports. 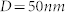; 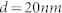; 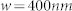; 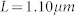.
